# Reovirus activated NK cells show enhanced cetuximab mediated antibody-dependent cellular cytotoxicity against colorectal cancer cells

**DOI:** 10.1186/2051-1426-3-S2-P340

**Published:** 2015-11-04

**Authors:** Xing Zhao, Narendiran Rajasekaran, Cariad Chester, Atsushi Yonezawa, Suparna Dutt, Matt Coffey, Holbrook E Kohrt

**Affiliations:** 1 Stanford University, Stanford, CA, USA; 2 Oncolytics Biotech, Calagary, Canada

## Background

The naturally occurring oncolytic virus, reovirus, exhibits cytotoxic effects on cancer cells. Reovirus is safe, and currently is in multiple clinical trials testing for the treatment of different cancers. NK cells are innate immune effectors that mediate antibody dependent cellular cytotoxicity (ADCC) against tumor cells.

## Methods

Here we investigated the direct effect of reovirus on NK cell mediated ADCC against the EGFR (Epidermal Growth Factor) positive colorectal cancer cell line: DLD-1 (KRAS mut). NK cells isolated from human PBMCs were cultured with 1pfu of reovirus for 12 hrs. These reovirus treated NK cells were co-cultured with DLD-1 cells coated with increasing concentrations anti-EGFR antibody cetuximab. ADCC was measured after 4hrs using a lactate dehydrogenase (LDH) based cytotoxicity assay. We observed that the reovirus treated NK cells (Reo-NK cells) exhibited a ~16-fold increase in cytotoxicity against DLD-1 (16.3% ±1.5, n=3) compared to untreated NK cells (NK cells), even in the absence of any cetuximab antibody. In the presence of cetuximab antibody, NK cells showed a dose dependent increase in ADCC, with maximum ADCC, observed at 0.1 µg/ml of cetuximab (DLD-NK: 33.4%± 7.1, n=3). Interestingly, Reo-NK cells showed maximum ADCC even at 0.01 µg/ml of cetuximab (DLD1-Reo-NK: 39.1±7.4, DLD1-NK: 26.7±2.4%, n=3).

## Results

To further investigate the factors contributing to the increased cytotoxic potential of Reo-NK cells we performed flow cytometry analysis to determine the expression of activation and degranulation markers on NK cells. We observed that in presence of tumor cells Reo-NK cells exhibited a 2-fold increased expression of activation marker CD69 when compared to untreated NK cells (Reo-NK-70.4%, NK-35.2%). There was a 3-fold increase in expression of CD107a on Reo-NK cells when compared to NK cells (Reo-NK: 14.6%; NK: 4.45%). Further, treating Reo-NK and NK cells with concanamycin A (CMA), an inhibitor of perforin, reduced Reo-NK cell mediated ADCC by 3.17 fold (CMA treated-28.2%, CMA untreated 18.9%) indicating a perforin-mediated cytotoxicity contributes to the increased cytotoxicity of Reo-NK cells. Interestingly, UV attenuated reovirus failed to increase activation and ADCC of NK cells.

## Conclusions

Thus, our results demonstrate that reovirus treatement activated NK cells, and lowered the threshold of cetuximab required to achieve maximum ADCC. We propose that reovirus activated NK cells are a potential candidate for cell based immunotherapy in combination with FDA approved tumor targeting antibodies. Further studies are ongoing to investigate the underlying mechanisms that contribute to the increase in cytotoxicity by NK cells treated with reovirus.

